# The crystal structure of 1-(2-iodo­benzo­yl)-4-(pyrimidin-2-yl)piperazine: a three-dimensional hydrogen-bonded framework, augmented by π–π stacking inter­actions and I⋯N halogen bonds

**DOI:** 10.1107/S205698901801811X

**Published:** 2019-01-04

**Authors:** Ninganayaka Mahesha, Hemmige S. Yathirajan, Tetsundo Furuya, Takashiro Akitsu, Christopher Glidewell

**Affiliations:** a Department of Studies in Chemistry, University of Mysore, Manasagangotri, Mysuru-570 006, India; b Department of Chemistry, Faculty of Science, Tokyo University of Science, 1-3 Kagurazaka, Shinjuku-ku, Tokyo 162-8601, Japan; c School of Chemistry, University of St Andrews, St Andrews, Fife KY16 9ST, UK

**Keywords:** pyrimidine, piperazine, crystal structure, mol­ecular conformation, hydrogen bonding, supra­molecular assembly

## Abstract

In 1-(2-iodo­benzo­yl)-4-(pyrimidin-2-yl)piperazine, C_15_H_15_IN_4_O, the central piperazine ring adopts an almost perfect chair conformation with the pyrimidine substituent in an equatorial site. The planar amide unit makes a dihedral angle of 80.44 (7)° with the phenyl ring. A combination of C—H⋯O and C—H⋯π(arene) hydrogen bonds links the mol­ecules into a complex three-dimensional network structure, augmented by a π–π stacking inter­action and an I⋯N halogen bond, all involving different pairs of inversion-related mol­ecules. Comparisons are made with the structures of a number of related compounds.

## Abstract 2

Mol­ecules of the title compound are linked into a complex three-dimensional network by a combination of C—H⋯O and C—H⋯π(arene) hydrogen bonds.

## Chemical context   

**Figure 1:**
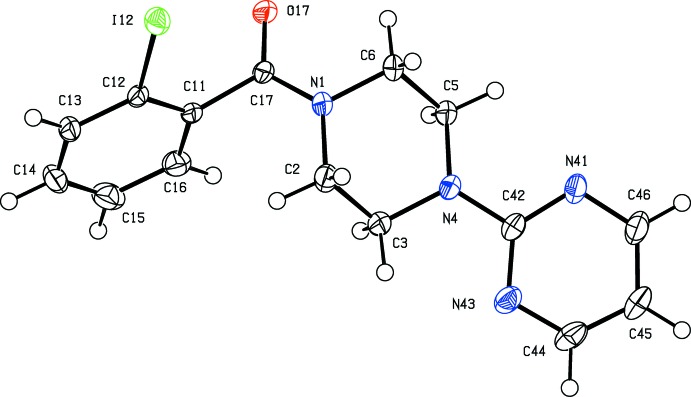
The mol­ecular structure of compound (I) showing the atom-labelling scheme. Displacement ellipsoids are drawn at the 50% probability level.

Pyrimidine derivatives are well represented amongst the range of heterocyclic compounds that exhibit a broad spectrum of biological activities such as analgesic and anti-inflammatory activity (Amin *et al.*, 2009 ▸), anti­bacterial (Kuyper *et al.*, 1996 ▸), anti­depressant (Kim *et al.*, 2010 ▸), anti­microbial and anti-oxidant (Padmaja *et al.*, 2009 ▸) and anti­viral activities (Ibrahim & El-Metwally, 2010 ▸). Piperazine-based compounds also exhibit anti-cancer properties (Abdel-Jalil *et al.*, 2005 ▸), while the combination of pyrimidine and piperazine units is found in bu­spirone, 8-[4-(4-pyrimidin-2-ylpiperazin-1-yl)but­yl]-8-aza­spiro­[4.5]decane-7,9-dione (Tollefson *et al.*, 1991 ▸), which can be used in the treatment of anxiety. With these considerations in mind, we have now synthesized the title compound (I) (Fig. 1 ▸), and we report here its mol­ecular and supra­molecular structure. Compound (I) was prepared by reaction of 1-(2-pyrimid­yl)piperazine with 2-iodo­benzoic acid in the presence of di­methyl­amino­prop­yl)-3-ethyl­carbodimide as the dehydrating agent.
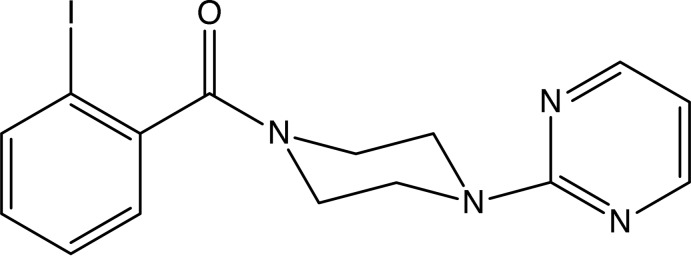


## Structural commentary   

Within the mol­ecule of compound (I) (Fig. 1 ▸), the piperazine ring adopts an almost perfect chair conformation. The ring-puckering parameters, calculated for the atom sequence (N1, C2, C3, N4, C5, C6) are *Q* = 0.557 (3) Å, θ = 1.2 (3) ° and φ = 258 (14)°, while for an idealized chair form the value of θ is 0.0° (Boeyens, 1978 ▸). The pyrimidine substituent at the pyramidal atom N4 occupies an equatorial site, but the amidic unit at atom N1 is effectively planar, and the r.m.s. deviation from the mean plane of atoms (C2, N1, C6, C17, O17 and C11) is only 0.027 Å. The dihedral angle between this plane and the aryl ring (C11–C16) is 80.44 (7)°. The mol­ecules of (I) thus exhibit no inter­nal symmetry and so are conformationally chiral, as confirmed by the centrosymmetric space group in which the molecule crystallizes.

## Supra­molecular features   

The supra­molecular assembly of compound (I) is built from two C—H⋯O hydrogen bonds, involving the aryl and pyrimidyl atoms C16 and C45 as the donors (Table 1 ▸), and one C—H⋯π(arene) hydrogen bond: there is a further inter­molecular C—H⋯O contact, involving atom C13, but here the *D*—H⋯*A* angle is less than 140°, and so this contact cannot be regarded as structurally significant (Wood *et al.*, 2009 ▸). There are also present in the structure a π–π stacking inter­action between pairs of pyrimidine rings and an I⋯N halogen bond.

**Figure 2:**
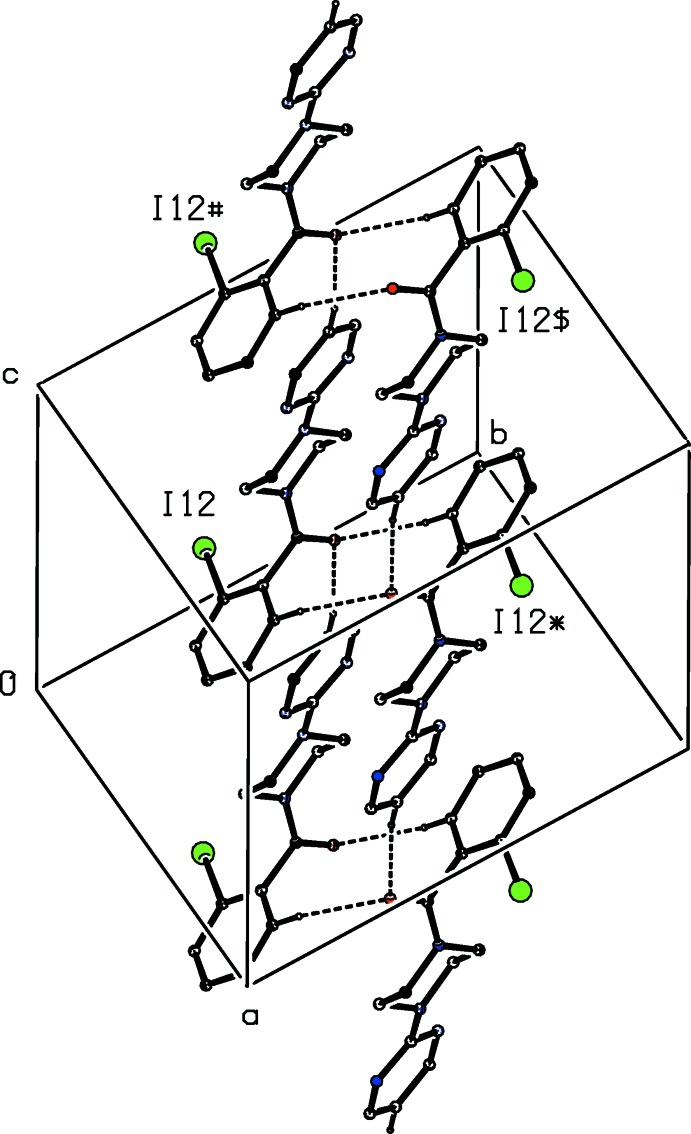
Part of the crystal structure of compound (I) showing the formation of a hydrogen-bonded chain of edge-fused rings parallel to the [001] direction. Hydrogen bonds are drawn as dashed lines and, for the sake of clarity, the H atoms not involved in the motif shown have been omitted. The I atoms marked with an asterisk (*), a hash (#) or a dollar sign ($) are at the symmetry positions (1 − *x*, 1 − *y*, 1 − *z*), (*x*, *y*, 1 + *z*) and (1 − *x*, 1 − *y*, 2 − *z*), respectively.

The hydrogen bonds give rise to a three-dimensional network structure of considerable complexity, but this is readily analysed in terms of three one-dimensional sub-structures (Ferguson *et al.*, 1998**a* ▸,b* ▸; Gregson *et al.*, 2000 ▸). The action of the two C—H⋯O hydrogen bonds in combination links mol­ecules related by inversion and translation into a chain of edge-fused rings running parallel to the [001] direction (Fig. 2 ▸), in which 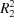
(10) (Etter, 1990 ▸; Etter *et al.*, 1990 ▸; Bernstein *et al.*, 1995 ▸) rings centred at (0.5, 0.5, *n* + 0.5) alternate with 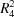
(28) rings centred at (0.5, 0.5, *n*), where *n* represents an integer in each case.

**Figure 3:**
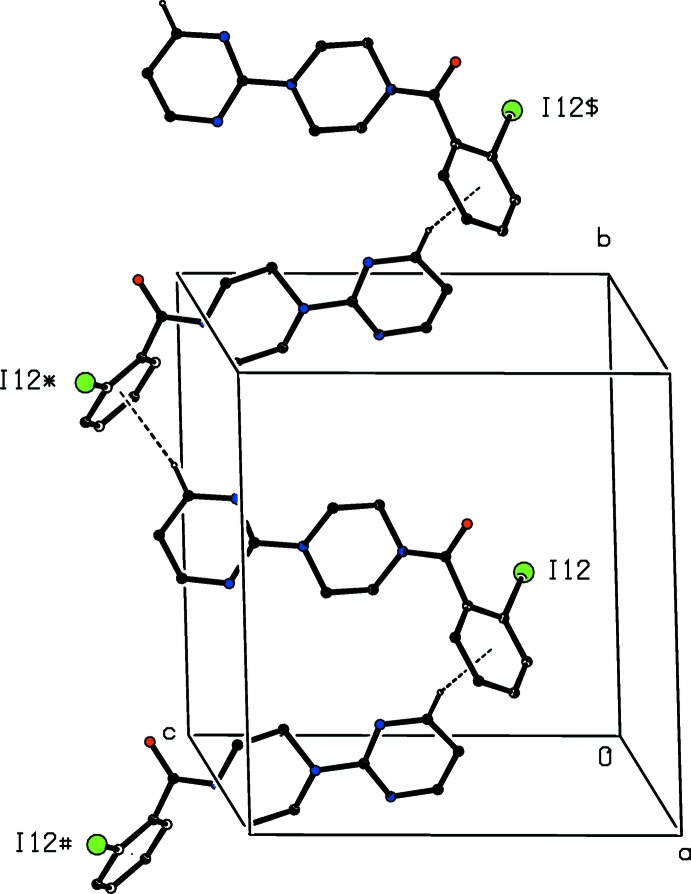
Part of the crystal structure of compound (I) showing the formation of a hydrogen-bonded chain parallel to the [010] direction. Hydrogen bonds are drawn as dashed lines and, for the sake of clarity, the H atoms not involved in the motif shown have been omitted. The I atoms marked with an asterisk (*), a hash (#) or a dollar sign ($) are at the symmetry positions (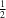 − *x*, 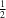 + *y*, 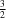 − *z*), (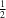 − *x*, −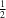 + *y*, 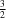 − *z*) and (*x*, 1 + *y*, *z*), respectively.

**Figure 4:**
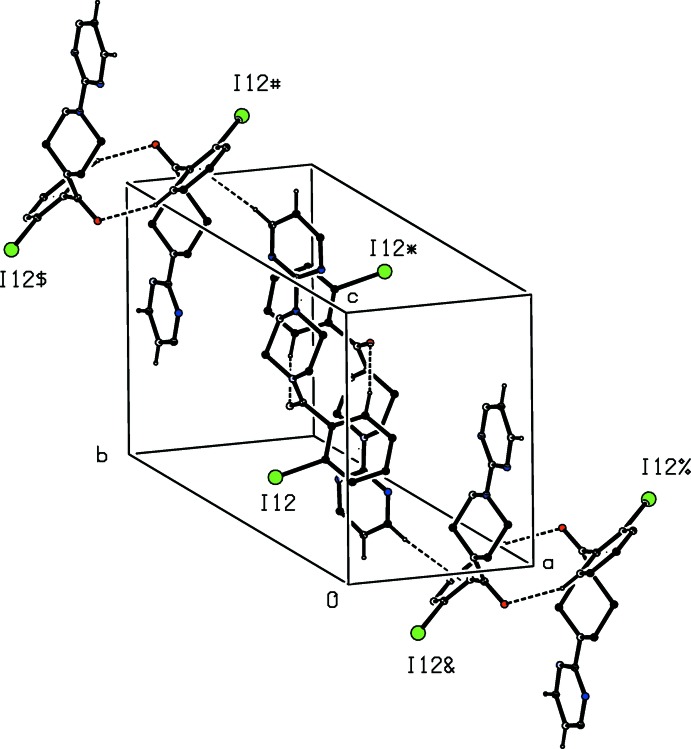
Part of the crystal structure of compound (I) showing the formation of a hydrogen-bonded chain of rings parallel to the [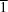
11] direction. Hydrogen bonds are drawn as dashed lines and, for the sake of clarity, the H atoms not involved in the motif shown have been omitted. The I atoms marked with an asterisk (*), a hash (#), a dollar sign ($), an ampersand (&) or a percent sign (%) are at the symmetry positions (1 − *x*, 1 − *y*, 1 − *z*), (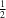 − *x*, 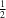 + *y*, 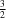 − *z*), (−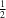 + *x*, 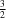 − *y*, 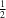 + *z*), (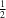 + *x*, 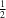 − *y*, −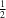 + *z*) and (1.5 − *x*, −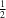 + *y*, 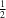 − *z*) respectively.

A second sub-structure can be identified in which the C—H⋯π(arene) hydrogen bond links mol­ecules related by a 2_1_ screw axis to form a chain running parallel to the [010] direction (Fig. 3 ▸). The chains parallel to [010] and [001] each use only one type of hydrogen bond, but the alternating action of the C—H⋯O and C—H⋯π(arene) hydrogen bonds involving atoms C16 and C46 as the donors (Table 1 ▸) links the mol­ecules into a chain of rings running parallel to the [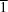
11] direction (Fig. 4 ▸). The combination of chains running parallel to [010], [001] and [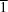
11] suffices to generate a continuous three-dimensional network structure.

**Figure 5:**
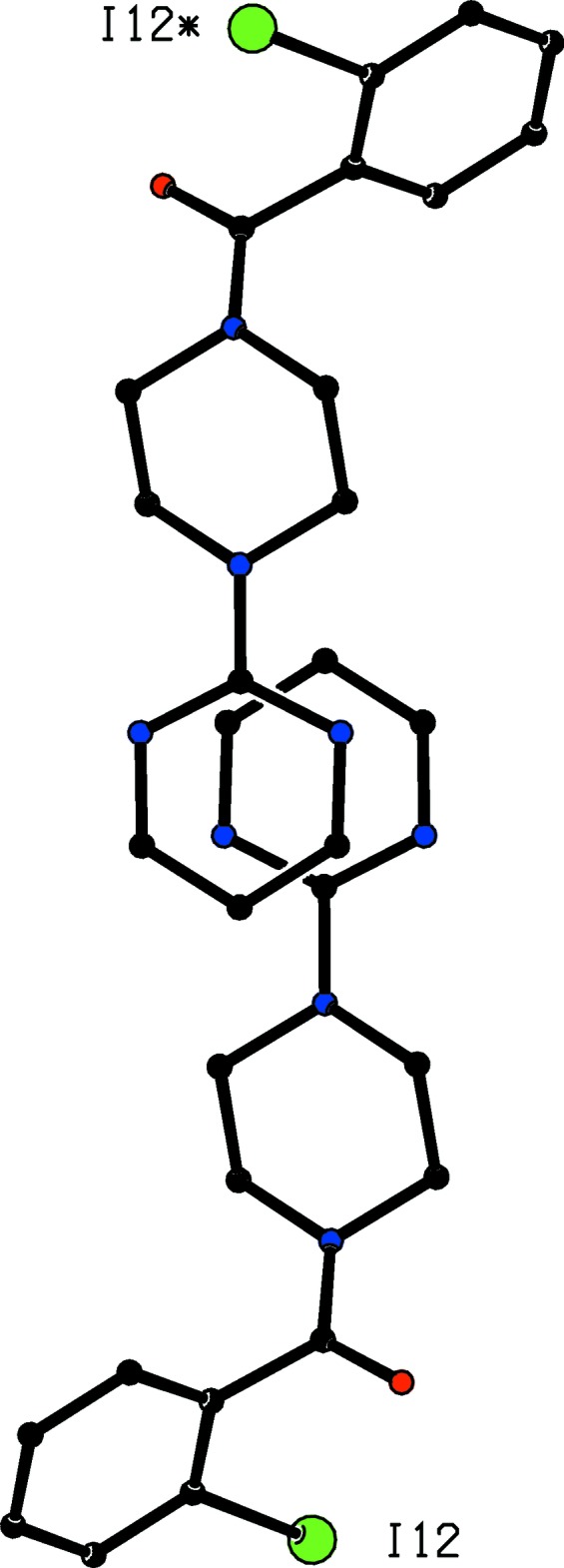
Part of the crystal structure of compound (I) showing the π–π stacking inter­action between adjacent pyrimidine rings. For the sake of clarity, the unit-cell outline and the H atoms have been omitted. The I atom marked with an asterisk (*) is at the symmetry position (1 − *x*, 1 − *y*, 2 − *z*).

The formation of the hydrogen-bonded network is augmented by two further inter­molecular inter­actions, each of which involves inversion related pairs of mol­ecules. The pyrimidine rings of the mol­ecules at (*x*, *y*, *z*) and (1 − *x*, 1 − *y*, 2 − *z*), which are components of the hydrogen-bonded chain along [001], are strictly parallel with an inter­planar spacing of 3.4295 (10) Å and a ring-centroid separation of 3.4924 (6) Å, thus giving rise to a π–π stacking inter­action (Fig. 5 ▸). Finally, we note a short inter­molecular I⋯N contact with geometrical parameters of I12⋯N41^i^ = 3.168 (2) Å and C12—I12⋯N41^i^ 174.83 (7)° [symmetry code: (i) −*x*, 1 − *y*, 1 − *z*], which can be regarded as a halogen bond (Gilday *et al.*, 2015 ▸; Cavallo *et al.*, 2016 ▸).

## Database survey   

**Figure 6:**
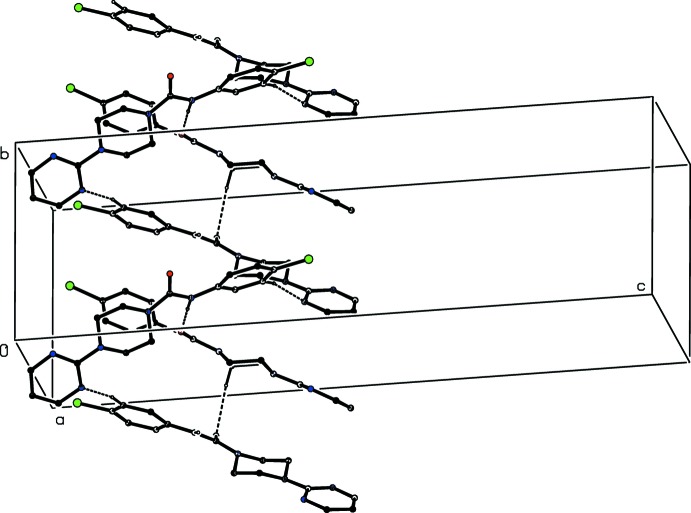
Part of the crystal structure of compound (III) showing the formation of a hydrogen-bonded chain parallel to [010]. The original atomic coordinates (Li, 2011*b* ▸) have been used and, for the sake of clarity, the H atoms not involved in the motif shown have been omitted.

**Figure 7:**
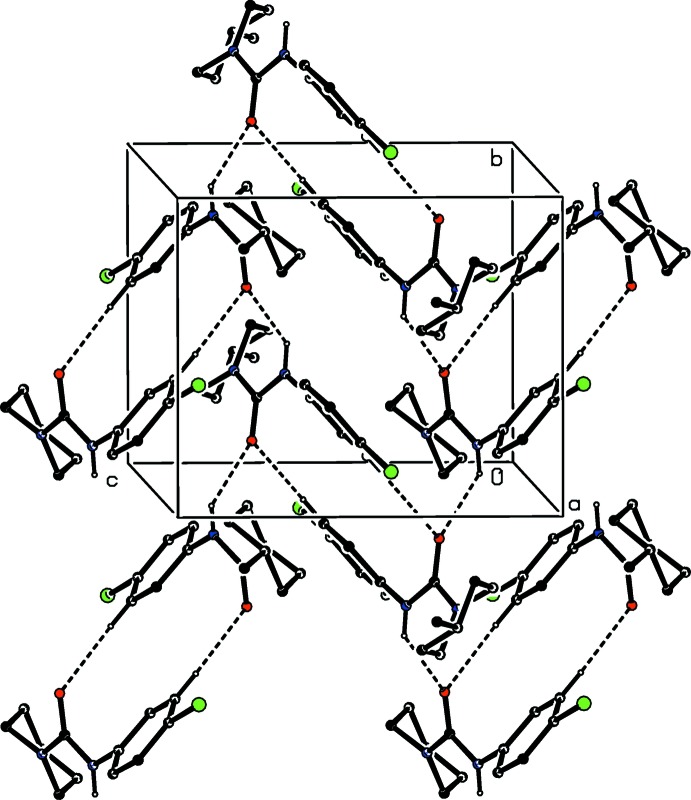
Part of the crystal structure of compound (IV) showing the formation of a hydrogen-bonded sheet parallel to (100). The original atomic coordinates (Li, 2011*a* ▸) have been used and, for the sake of clarity, the H atoms not involved in the motif shown have been omitted.

It is of inter­est briefly to compare the structure of compound (I) reported here with those of some related structures which have been recently reported. In 2-{4-[(1,3-benzodioxol-5-yl)meth­yl]piperazin-1-yl}pyrimidine (II), the mol­ecules are linked into sheets by a combination of C—H⋯π(arene) and C—H⋯π(pyrimidine) hydrogen bonds (Wu *et al.*, 2013 ▸). *N*-(4-Chloro­phen­yl)-4-(pyrimidin-2-yl)piperazine-1-carboxamide (III) crystallizes with *Z*′ = 2 in space group *P*2_1_/*c* (Li, 2011*b* ▸), and the mol­ecules are linked into chains by two independent N—H⋯O hydrogen bonds: these chains, parallel to [100], are of the 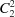
(8) type rather than of the *C*(4) type as originally reported. However, the original report overlooked the presence of C—H⋯O hydrogen bonds which, in combin­ation with the N—H⋯O hydrogen bond within the selected asymmetric unit, generates a second chain, this time running parallel to the [010] direction (Fig. 6 ▸), so that overall the supra­molecular assembly takes the form of a sheet parallel to (001). In the simpler analogue *N*-(4-chloro­phen­yl)-4-methyl­piperidine-1-carboxamide (IV), the assembly was reported (Li, 2011*a* ▸) as consisting of simple *C*(4) chains built from N—H⋯O hydrogen bonds. However, the presence in (IV) of a C—H⋯O hydrogen bond was overlooked, and the two hydrogen bonds together generate a complex sheet structure lying parallel to (100) (Fig. 7 ▸). Finally, we note also the structures of a number of salts of the 4-(pyrimidin-2-yl)piperazin-1-ium cation, including the chloride and nitrate (Yamuna *et al.*, 2014*a* ▸), the hydrogenfumarate (Yamuna *et al.*, 2014*b* ▸) and the butano­ate (Yamuna *et al.*, 2014*c* ▸).

## Synthesis and crystallization   

1-(2-Pyrimid­yl)piperazine was purchased from Sigma–Aldrich. For the synthesis of compound (I), 1-(3-di­methyl­amino­prop­yl)-3-ethyl­carbodimide (52 mg, 0.6 mmol), 1-hy­droxy­benzotriazole (81 mg, 0.6 mmol) and tri­ethyl­amine (0.5 ml, 1.8 mmol) were added to a solution of 2-iodo­benzoic acid (0.6 mmol) in *N*,*N*-di­methyl­formamide (5 ml) and the resulting mixture stirred for 20 mins at 273 K. A solution of 1-(2-pyrimid­yl)piperazine (100 mg, 0.6 mmol) in *N*,*N*-di­methyl­formamide (5 ml) was then added and stirring was continued overnight at ambient temperature. The reaction was confirmed to be complete using thin-layer chromatography, and the mixture was then quenched with water (10 ml) and extracted with ethyl acetate (20 ml). The organic layer was separated and washed successively with an aqueous hydro­chloric acid solution (1 mol dm^−3^), a saturated solution of sodium hydrogencarbonate and then with brine. The organic phase was dried over anhydrous sodium sulfate and the solvent was removed under reduced pressure. Crystals suitable for single-crystal X-ray diffraction were grown by slow evaporation, at ambient temperature and in the presence of air, of a solution in methanol; m. p. 450–452 K.

## Refinement   

Crystal data, data collection and structure refinement details are summarized in Table 2 ▸. All H atoms were located in difference maps, and they were subsequently treated as riding atoms in geometrically idealized positions with C—H distances of 0.95 Å (aromatic) or 0.99 Å (CH_2_), and with *U*_iso_(H) = 1.2*U*_eq_(C).

## Supplementary Material

Crystal structure: contains datablock(s) global, I. DOI: 10.1107/S205698901801811X/wm5479sup1.cif

Structure factors: contains datablock(s) I. DOI: 10.1107/S205698901801811X/wm5479Isup2.hkl

Supporting information file. DOI: 10.1107/S205698901801811X/wm5479Isup3.cml

CCDC reference: 1886570

Additional supporting information:  crystallographic information; 3D view; checkCIF report

## Figures and Tables

**Table 1:** Hydrogen-bond geometry (Å, °) # *Cg*1 is the centroid of the C11–C16 ring.

*D*—H⋯*A*	*D*—H	H⋯*A*	*D*⋯*A*	*D*—H⋯*A*
C16—H16⋯O17^i^	0.95	2.50	3.395 (3)	157
C45—H45⋯O17^ii^	0.95	2.57	3.508 (3)	169
C46—H46⋯*Cg*1^iii^	0.95	2.72	3.596 (3)	153

Symmetry codes: (i) 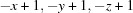; (ii) 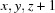; (iii) 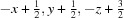.

**Table 2:** Experimental details

Crystal data	
Chemical formula	C_15_H_15_IN_4_O
*M*_r_	394.21
Crystal system, space group	Monoclinic, *P*2_1_/*n*
Temperature (K)	173
*a*, *b*, *c* (Å)	9.6417 (17), 13.604 (2), 12.174 (2)
β (°)	105.155 (2)
*V* (Å^3^)	1541.3 (4)
*Z*	4
Radiation type	Mo *K*α
μ (mm^−1^)	2.08
Crystal size (mm)	0.67 × 0.56 × 0.16

Data collection	
Diffractometer	Bruker APEXII CCD
Absorption correction	Multi-scan (*SADABS*; Bruker, 2015 ▸)
*T*_min_, *T*_max_	0.345, 0.717
No. of measured, independent and observed [*I* > 2σ(*I*)] reflections	8152, 3452, 3188
*R*_int_	0.067
(sin θ/λ)_max_ (Å^−1^)	0.652

Refinement	
*R*[*F* ^2^ > 2σ(*F* ^2^)], *wR*(*F* ^2^), *S*	0.028, 0.075, 1.05
No. of reflections	3452
No. of parameters	190
H-atom treatment	H-atom parameters constrained
Δρ_max_, Δρ_min_ (e Å^−3^)	0.96, −0.74

Computer programs: *APEX2* (Bruker, 2004 ▸), *SAINT* (Bruker, 2015 ▸), *SHELXT* (Sheldrick, 2015*a* ▸), *SHELXL2014* (Sheldrick, 2015*b* ▸) and *PLATON* (Spek, 2009 ▸).

## supplementary crystallographic information

### Crystal data

**Table:**

C_15_H_15_IN_4_O	*F*(000) = 776
*M**_r_* = 394.21	*D*_x_ = 1.699 Mg m^−^^3^
Monoclinic, *P*2_1_/*n*	Mo *K*α radiation, λ = 0.71073 Å
*a* = 9.6417 (17) Å	Cell parameters from 3152 reflections
*b* = 13.604 (2) Å	θ = 2.3–27.6°
*c* = 12.174 (2) Å	µ = 2.08 mm^−^^1^
β = 105.155 (2)°	*T* = 173 K
*V* = 1541.3 (4) Å^3^	Plate, colour
*Z* = 4	0.67 × 0.56 × 0.16 mm

### Data collection

**Table:**

Bruker APEXII CCD diffractometer	3452 independent reflections
Radiation source: fine focus sealed tube	3188 reflections with *I* > 2σ(*I*)
Graphite monochromator	*R*_int_ = 0.067
Detector resolution: 0.3333 pixels mm^-1^	θ_max_ = 27.6°, θ_min_ = 2.3°
φ and ω scans	*h* = −12→10
Absorption correction: multi-scan (*SADABS*; Bruker, 2015)	*k* = −17→16
*T*_min_ = 0.345, *T*_max_ = 0.717	*l* = −15→15
8152 measured reflections	

### Refinement

**Table:**

Refinement on *F*^2^	0 restraints
Least-squares matrix: full	Hydrogen site location: inferred from neighbouring sites
*R*[*F*^2^ > 2σ(*F*^2^)] = 0.028	H-atom parameters constrained
*wR*(*F*^2^) = 0.075	*w* = 1/[σ^2^(*F*_o_^2^) + (0.0235*P*)^2^ + 0.7279*P*] where *P* = (*F*_o_^2^ + 2*F*_c_^2^)/3
*S* = 1.05	(Δ/σ)_max_ = 0.001
3452 reflections	Δρ_max_ = 0.96 e Å^−^^3^
190 parameters	Δρ_min_ = −0.74 e Å^−^^3^

### Special details

**Table:**

Geometry. All esds (except the esd in the dihedral angle between two l.s. planes) are estimated using the full covariance matrix. The cell esds are taken into account individually in the estimation of esds in distances, angles and torsion angles; correlations between esds in cell parameters are only used when they are defined by crystal symmetry. An approximate (isotropic) treatment of cell esds is used for estimating esds involving l.s. planes.

### Fractional atomic coordinates and isotropic or equivalent isotropic displacement parameters (Å^2^)

**Table:**

	*x*	*y*	*z*	*U*_iso_*/*U*_eq_	
N1	0.2388 (2)	0.44984 (14)	0.52539 (16)	0.0253 (4)	
C2	0.2263 (3)	0.36447 (15)	0.5950 (2)	0.0282 (6)	
H2A	0.2567	0.3046	0.5613	0.034*	
H2B	0.1248	0.3559	0.5964	0.034*	
C3	0.3195 (3)	0.37777 (17)	0.7159 (2)	0.0290 (6)	
H3A	0.3033	0.3225	0.7640	0.035*	
H3B	0.4222	0.3777	0.7159	0.035*	
N4	0.2839 (2)	0.47043 (14)	0.76229 (16)	0.0260 (4)	
C5	0.2977 (3)	0.55610 (16)	0.69257 (18)	0.0247 (5)	
H5A	0.3991	0.5637	0.6904	0.030*	
H5B	0.2689	0.6163	0.7265	0.030*	
C6	0.2028 (3)	0.54263 (17)	0.57233 (19)	0.0273 (5)	
H6A	0.1005	0.5421	0.5735	0.033*	
H6B	0.2174	0.5980	0.5239	0.033*	
C17	0.2929 (3)	0.45026 (16)	0.43404 (17)	0.0211 (4)	
O17	0.3118 (2)	0.52552 (12)	0.38448 (14)	0.0334 (4)	
C11	0.3328 (3)	0.35268 (15)	0.39109 (19)	0.0195 (5)	
C12	0.2434 (2)	0.30692 (15)	0.29625 (17)	0.0191 (4)	
I12	0.03709 (2)	0.36186 (2)	0.21850 (2)	0.02547 (8)	
C13	0.2883 (3)	0.22226 (16)	0.25098 (19)	0.0257 (5)	
H13	0.2268	0.1909	0.1867	0.031*	
C14	0.4237 (3)	0.18428 (17)	0.3008 (2)	0.0327 (6)	
H14	0.4553	0.1269	0.2699	0.039*	
C15	0.5130 (3)	0.22889 (19)	0.3947 (2)	0.0346 (6)	
H15	0.6054	0.2021	0.4284	0.042*	
C16	0.4677 (3)	0.31314 (18)	0.4401 (2)	0.0279 (5)	
H16	0.5293	0.3438	0.5048	0.033*	
N41	0.2769 (2)	0.57158 (16)	0.91400 (17)	0.0301 (5)	
C42	0.3025 (2)	0.48172 (17)	0.87784 (18)	0.0221 (4)	
N43	0.3414 (2)	0.40176 (15)	0.94349 (17)	0.0280 (4)	
C44	0.3528 (3)	0.4150 (2)	1.0545 (2)	0.0328 (6)	
H44	0.3790	0.3601	1.1039	0.039*	
C45	0.3288 (3)	0.5033 (2)	1.1012 (2)	0.0318 (6)	
H45	0.3381	0.5108	1.1804	0.038*	
C46	0.2902 (3)	0.5804 (2)	1.0258 (2)	0.0338 (6)	
H46	0.2721	0.6427	1.0546	0.041*	

### Atomic displacement parameters (Å^2^)

**Table:**

	*U*^11^	*U*^22^	*U*^33^	*U*^12^	*U*^13^	*U*^23^
N1	0.0418 (12)	0.0171 (8)	0.0180 (9)	0.0026 (9)	0.0095 (8)	−0.0025 (7)
C2	0.0455 (17)	0.0190 (11)	0.0247 (13)	−0.0072 (10)	0.0172 (12)	−0.0029 (8)
C3	0.0496 (17)	0.0193 (10)	0.0211 (12)	0.0030 (12)	0.0149 (11)	0.0016 (9)
N4	0.0421 (12)	0.0213 (9)	0.0161 (9)	0.0009 (10)	0.0101 (8)	−0.0012 (7)
C5	0.0377 (13)	0.0191 (10)	0.0182 (11)	0.0013 (11)	0.0088 (9)	−0.0022 (8)
C6	0.0411 (14)	0.0223 (10)	0.0181 (10)	0.0059 (11)	0.0069 (10)	−0.0043 (9)
C17	0.0304 (11)	0.0176 (9)	0.0124 (9)	0.0005 (10)	0.0007 (8)	−0.0020 (8)
O17	0.0646 (13)	0.0173 (7)	0.0196 (8)	0.0013 (9)	0.0133 (8)	0.0014 (6)
C11	0.0302 (12)	0.0145 (9)	0.0150 (10)	−0.0006 (9)	0.0079 (9)	0.0014 (7)
C12	0.0263 (11)	0.0174 (9)	0.0153 (9)	−0.0017 (9)	0.0084 (8)	0.0003 (8)
I12	0.02670 (12)	0.02407 (11)	0.02409 (11)	−0.00088 (5)	0.00387 (8)	−0.00347 (5)
C13	0.0421 (14)	0.0163 (10)	0.0218 (11)	−0.0010 (11)	0.0141 (10)	−0.0016 (8)
C14	0.0491 (16)	0.0192 (11)	0.0374 (14)	0.0070 (12)	0.0250 (12)	0.0005 (10)
C15	0.0345 (13)	0.0304 (13)	0.0408 (14)	0.0108 (13)	0.0132 (11)	0.0083 (11)
C16	0.0313 (12)	0.0261 (11)	0.0238 (11)	0.0009 (11)	0.0031 (9)	0.0020 (9)
N41	0.0378 (12)	0.0343 (11)	0.0186 (10)	0.0082 (10)	0.0081 (8)	−0.0043 (8)
C42	0.0214 (10)	0.0286 (11)	0.0174 (10)	−0.0028 (10)	0.0070 (8)	−0.0009 (9)
N43	0.0368 (12)	0.0279 (10)	0.0214 (10)	−0.0055 (10)	0.0115 (8)	0.0019 (8)
C44	0.0384 (14)	0.0388 (14)	0.0224 (12)	−0.0092 (13)	0.0099 (10)	0.0063 (10)
C45	0.0351 (13)	0.0452 (14)	0.0169 (11)	−0.0073 (13)	0.0098 (9)	−0.0023 (10)
C46	0.0400 (14)	0.0428 (14)	0.0199 (12)	0.0034 (13)	0.0102 (10)	−0.0060 (11)

### Geometric parameters (Å, º)

**Table:**

N1—C17	1.346 (3)	C11—C12	1.394 (3)
N1—C2	1.461 (3)	C12—C13	1.393 (3)
N1—C6	1.464 (3)	C12—I12	2.104 (2)
C2—C3	1.521 (4)	C13—C14	1.388 (4)
C2—H2A	0.9900	C13—H13	0.9500
C2—H2B	0.9900	C14—C15	1.379 (4)
C3—N4	1.459 (3)	C14—H14	0.9500
C3—H3A	0.9900	C15—C16	1.391 (3)
C3—H3B	0.9900	C15—H15	0.9500
N4—C42	1.379 (3)	C16—H16	0.9500
N4—C5	1.468 (3)	N41—C46	1.339 (3)
C5—C6	1.521 (3)	N41—C42	1.344 (3)
C5—H5A	0.9900	C42—N43	1.344 (3)
C5—H5B	0.9900	N43—C44	1.340 (3)
C6—H6A	0.9900	C44—C45	1.375 (4)
C6—H6B	0.9900	C44—H44	0.9500
C17—O17	1.226 (3)	C45—C46	1.380 (4)
C17—C11	1.513 (3)	C45—H45	0.9500
C11—C16	1.390 (3)	C46—H46	0.9500
			
C17—N1—C2	126.25 (18)	C16—C11—C12	119.3 (2)
C17—N1—C6	120.10 (19)	C16—C11—C17	119.1 (2)
C2—N1—C6	113.23 (17)	C12—C11—C17	121.3 (2)
N1—C2—C3	110.4 (2)	C13—C12—C11	120.5 (2)
N1—C2—H2A	109.6	C13—C12—I12	118.05 (18)
C3—C2—H2A	109.6	C11—C12—I12	121.41 (16)
N1—C2—H2B	109.6	C14—C13—C12	119.2 (2)
C3—C2—H2B	109.6	C14—C13—H13	120.4
H2A—C2—H2B	108.1	C12—C13—H13	120.4
N4—C3—C2	109.7 (2)	C15—C14—C13	120.7 (2)
N4—C3—H3A	109.7	C15—C14—H14	119.6
C2—C3—H3A	109.7	C13—C14—H14	119.6
N4—C3—H3B	109.7	C14—C15—C16	119.9 (2)
C2—C3—H3B	109.7	C14—C15—H15	120.0
H3A—C3—H3B	108.2	C16—C15—H15	120.0
C42—N4—C3	120.7 (2)	C11—C16—C15	120.3 (2)
C42—N4—C5	119.60 (18)	C11—C16—H16	119.8
C3—N4—C5	113.33 (17)	C15—C16—H16	119.8
N4—C5—C6	109.75 (19)	C46—N41—C42	116.0 (2)
N4—C5—H5A	109.7	N41—C42—N43	126.0 (2)
C6—C5—H5A	109.7	N41—C42—N4	116.7 (2)
N4—C5—H5B	109.7	N43—C42—N4	117.3 (2)
C6—C5—H5B	109.7	C44—N43—C42	115.3 (2)
H5A—C5—H5B	108.2	N43—C44—C45	123.8 (2)
N1—C6—C5	109.6 (2)	N43—C44—H44	118.1
N1—C6—H6A	109.8	C45—C44—H44	118.1
C5—C6—H6A	109.8	C44—C45—C46	115.8 (2)
N1—C6—H6B	109.8	C44—C45—H45	122.1
C5—C6—H6B	109.8	C46—C45—H45	122.1
H6A—C6—H6B	108.2	N41—C46—C45	123.1 (2)
O17—C17—N1	123.3 (2)	N41—C46—H46	118.5
O17—C17—C11	118.59 (19)	C45—C46—H46	118.5
N1—C17—C11	118.07 (19)		
			
C17—N1—C2—C3	116.2 (3)	C17—C11—C12—I12	−7.2 (3)
C6—N1—C2—C3	−56.4 (3)	C11—C12—C13—C14	−0.6 (3)
N1—C2—C3—N4	54.1 (3)	I12—C12—C13—C14	−179.83 (16)
C2—C3—N4—C42	151.8 (2)	C12—C13—C14—C15	0.6 (3)
C2—C3—N4—C5	−56.4 (3)	C13—C14—C15—C16	−0.3 (4)
C42—N4—C5—C6	−150.6 (2)	C12—C11—C16—C15	0.1 (3)
C3—N4—C5—C6	57.3 (3)	C17—C11—C16—C15	−173.4 (2)
C17—N1—C6—C5	−116.3 (2)	C14—C15—C16—C11	−0.1 (4)
C2—N1—C6—C5	56.8 (3)	C46—N41—C42—N43	−1.0 (4)
N4—C5—C6—N1	−55.3 (2)	C46—N41—C42—N4	177.6 (2)
C2—N1—C17—O17	−173.7 (3)	C3—N4—C42—N41	175.3 (2)
C6—N1—C17—O17	−1.6 (4)	C5—N4—C42—N41	25.3 (3)
C2—N1—C17—C11	6.0 (4)	C3—N4—C42—N43	−6.0 (3)
C6—N1—C17—C11	178.1 (2)	C5—N4—C42—N43	−156.1 (2)
O17—C17—C11—C16	94.4 (3)	N41—C42—N43—C44	1.1 (4)
N1—C17—C11—C16	−85.3 (3)	N4—C42—N43—C44	−177.5 (2)
O17—C17—C11—C12	−78.9 (3)	C42—N43—C44—C45	−0.7 (4)
N1—C17—C11—C12	101.4 (3)	N43—C44—C45—C46	0.4 (4)
C16—C11—C12—C13	0.2 (3)	C42—N41—C46—C45	0.5 (4)
C17—C11—C12—C13	173.52 (19)	C44—C45—C46—N41	−0.3 (4)
C16—C11—C12—I12	179.44 (16)		

### Hydrogen-bond geometry (Å, º)

# Cg1 is the centroid of the C11–C16 ring.

**Table:**

*D*—H···*A*	*D*—H	H···*A*	*D*···*A*	*D*—H···*A*
C13—H13···O17^i^	0.95	2.40	3.160 (3)	136
C16—H16···O17^ii^	0.95	2.50	3.395 (3)	157
C45—H45···O17^iii^	0.95	2.57	3.508 (3)	169
C46—H46···*Cg*1^iv^	0.95	2.72	3.596 (3)	153

Symmetry codes: (i) −*x*+1/2, *y*−1/2, −*z*+1/2; (ii) −*x*+1, −*y*+1, −*z*+1; (iii) *x*, *y*, *z*+1; (iv) −*x*+1/2, *y*+1/2, −*z*+3/2.
